# Evaluation of the effect of follicular stimulating hormone on the in vitro bovine spermatogonial stem cells self-renewal: An experimental study

**Published:** 2017-12

**Authors:** Masoome Jabarpour, Parviz Tajik

**Affiliations:** *Department of Theriogenology, Faculty of Veterinary Medicine, University of Tehran, Tehran, Iran.*

**Keywords:** Sertoli cell, SSCs, FSH, Self-renewal

## Abstract

**Background:**

Spermatogonial stem cells (SSCs) are undifferentiated cells which are highly reproducible and expandable. Several studies have been conducted to reproduce these cells in culture. They used growth factors, hormones and different feeder cells to improve survival and proliferation of SSCs.

**Objective:**

This study was conducted to evaluate the effects of follicular stimulating hormone (FSH) on gene expression of fibroblast growth factor (FGF2) and glial cell-derived neurotrophic factor (GDNF) in Sertoli cells.

**Materials and Methods:**

Sertoli cells and SSCs were isolated from 3-5 month-old calves. Bovine testicular cells were cultured for 15 days with or without FSH. Identification of these cells was confirmed by immunocytochemistry analysis. Colony formation of SSCs was evaluated using an inverted microscope. The gene expression of FGF2 and GDNF and the gene markers bcl6b, thy-1, and C-kit were evaluated using the quantitative RT-PCR technique.

**Results:**

The results indicated that FSH increased colonization of SSCs. the expression of GDNF, FGF2, and markers of undifferentiated spermatogonia was increased following culture in control and FSH groups (p<0.05), this increase was more in FSH group. Conversely, the expression of C-kit was decreased in both groups (p<0.05).

**Conclusion:**

The results showed that FSH can increase the self-renewal of SSCs in vitro via upregulation of GDNF and FGF2 expression in Sertoli cells.

## Introduction

Spermatogenesis is a complex developmental process of cell proliferation and differentiation that originates from spermatogonial stem cells (SSCs) and produce mature spermatozoa (1). SSCs have two main features including self-renewing and differentiation properties, that maintains spermatogenesis (1). In this context thy1 (1-3) and bcl6b (3-5) are known undifferentiated markers of spermatogonia, whereas C-kit (6) considered as an SSCs differentiation marker. The balance between SSCs self-renewal and differentiation requires a specific microenvironment, called niche (1). In mammalian testes, Sertoli cells, basal membrane and, interstitial cells are the main components of SSCs niche (7). Sertoli cells generate growth factors required for self-renewing of SSCs such as glial cell derived neurotrophic factor (GDNF), Basic fibroblast growth factor (bFGF) and Kit (8-10). GDNF is a critical factor for self-generation of SSCs. GDNF is a protein secreted after generation of Sertoli cells and is responsible for maintaining and proliferating SSCs in both body and culture (9). Fibroblast growth factor 2 (FGF2) is secreted and expressed by Sertoli cells, Leydig cells and differentiated germ cells and stimulates self-renewing of SSCs (11). SSCs are undifferentiated cells which are highly reproducible and expandable. Biotechnologically, these cells are particularly important, because they are the only adult stem cells which can transfer genetic information to the next generation (12). To study characteristics and function of SSCs, it is essential to achieve a sufficient number of them (13). For this purpose, several studies have been conducted successfully to proliferation these cells in vitro. They have used growth factors, hormones and different feeder cells to improve survival, proliferation and occasionally differentiation of SSCs (14-17). Hormonal control of spermatogenesis is through follicle stimulating hormone (FSH) and testosterone activity on Sertoli cells. FSH is an essential prerequisite for maintaining spermatogenesis in adult mammals (18, 19). FSH stimulates GDNF production by the Sertoli cells and consequently increases the SSCs self-renewal (20). This study was conducted to evaluate the effect of FSH on the expression of the growth factors FGF2 and GDNF in Sertoli cells.

## Materials and methods

This experimental study was performed at Stem Cell Research Center, Faculty of Veterinary Medicine, University of Tehran between November 2015 and July 2016.

**Animals and testicular biopsy**

Testicular biopsies were obtained from 8 Holstein calves (4 calves were used for evaluation of colonization and 4 calves for gene expression) aged between 3 to 5 months, as previously described (21). Following testicular biopsy, samples taken from testicle were placed on ice and transferred to the laboratory within 2 hr, before they could be used for experiments. 

**Cell Isolation**

Cell isolation was implemented using a two-step enzymatic isolation procedure as previously used by our lab (22). Briefly, the obtained testis tissue was washed three times in DMEM containing antibiotics and Fetal Bovine Serum (FBS) 10% (Sigma, USA). They were then minced into small pieces as much as possible by a sterile scissor. The minced testicular tissue was suspended in DMEM containing 1 mg/ml collagenase (Sigma-Aldrich, USA), 1 mg/ml hyaluronidase (Sigma-Aldrich, USA), 1 mg/ml trypsin (Sigma-Aldrich, USA) and 5 µg/ml DNase (Fermentas, Germany) at 37°C in a shaker incubator operated at 80 cycles per minute for approximately 60 min. After three times washing in DMEM and removal the interstitial cells, during the second step of enzymatic digestion, the seminiferous tubules were again incubated at 37°C in DMEM containing 1 mg/ml collagenase, 1 mg/ml hyaluronidase and 5 µg/ml DNase. In this step, seminiferous tubules were deconstructed and their cells were separated. Finally, obtained cellular suspension was centrifuged at 30×g for 2 min to achieve population individual cells. Following filtration through 77 and 55 mm nylon filters, the cells were pelleted. The pellet was re-suspended in DMEM containing antibiotics and 10% fetal bovine serum (FBS).

**Cell culture**

Cell culture for colonization and gene expression were conducted as the procedure which was previously used by our lab (23). To assess colonization and gene expression 24-well and 6-well plates (TPP, Switzerland) were used, respectively. To evaluate the gene expression and colonization, cells were seeded at the concentrations of 10*105 and 3*104 per well contacting DMEM, respectively. 

The plates were incubated at 37oC in the presence of 5% CO_2_ for 15 days. DMEM (Sigma-Aldrich, St. Louis, MO, USA) containing 10% FBS, 4 mM L-glutamine, 0.1 mM non-essential amino acids, 100 IU/mL penicillin and 100 mg/mL streptomycin was used for culturing cells. The cells were cultured in two groups including group1 (Control) and group2 (FSH 30 IU/ml). Culture medium together with the FSH was refreshed every 3 days.

**Cells identification**

Vimentin is a cytoskeleton protein in Sertoli cell cytoplasm. At day 6 of culture, for Sertoli cells identification, Vimentin was stained, as described by Anway *et al* and Tajik *et al* (24, 25) and the specific marker Oct-4 was assessed in colonies of SSCs by the method proposed by Kubota *et al* (9).

**Colony assay**

Four cell populations from different calves were used for evaluation of colony formation. The colonization of SSCs in the control and FSH groups was assessed using an inverted microscope (IX71, Olympus, Japan).

**Gene expression assessment (qRT-PCR)**

Expression of the considered genes was assessed in the days 0, 6 and 12. Following trypsinization of the cultured cells (n=4 cell population from different calves), total RNA existing in the cells was extracted using Trizol reagent (Fermentas, Germany). In order to eliminate contamination of DNA, the extracted RNA was treated by DNase І (Fermentas, Germany). The concentration of the extracted RNA was determined by using spectrophotometry (Eppendorff, Germany). cDNAs were built by using 500ng RNA extracted and oligo-primers and cDNA synthesis kit (Fermentas, Germany).

Table I lists the primers of the considered genes. qRT-PCR was done by using SYBR Green mastermix (Fermentas, Germany) and by thermocycler (Applied Biosystems, USA). qRT-PCR started with a primary melting stage for 5 min at 95°C to activate polymerase and continued with 40 cycles including melting (30 s at 95°C), synthesis (30 s at 58°C) and formation (30 s at 72°C). Quality of qRT-PCR reactions was determined by melting curve analysis. 

For each sample, qRT-PCR was done for reference gene (β-actin) and target gene simultaneously. Cycle threshold (Ct) of the reference gene was subtracted from cycle threshold of the target gene to obtain ΔCt. In each nteraction interaction, Ct on time point 0 was considered as calibrator. Consequently, the relative gene expression was obtained by using Livak and Schmittgen (26) and calculation of ΔΔCt.

**Ethical consideration**

The research was conducted in accordance with guidelines of the Animal Ethics Committee at the University of Tehran.

**Statistical analysis**

All data were analyzed using Statistical Package for the Social Sciences, version 24.0, SPSS Inc, Chicago, Illinois, USA (SPSS). Data related to colonization and gene expression was assessed by using paired-samples *t*-test.

**Table I:** Antibodies introduction for SSC

**AB**	**Name**	**Concentration**	**Company**
Primary AB	Rabbit anti Oct 4	1/100	Abcam
Secondary AB	Goat anti rabbit IGg	1/100	Abcam

**Table II:** Antibodies introduction for Sertoli cells

**AB**	**Name**	**Concentration**	**Company**
Primary AB	Mouse anti vimentin	1/200	Abcam
Secondary AB	Sheep anti-mouse IGg	1/100	Abcam

**Table III:** Sequence of the primers used for qRT-PCR

**Gene**	**Forward primer (3′-5′)**	**Reverse primer (5′-3′)**
B-ACTIN	TCG CCC GAG TCC ACA CAG	ACC TCA ACC CGC TCC CAA G
FGF2	AAA ACA GGA CCT GGG CAG AA	ATA TAC CTC TTC ATG TAA AAT GAG ATC AGA TG
GDNF	GCAGCC GAA ACA ATG TAC GA	AAG GCG ATG GGT CTG CAA
THY-1	TTC ATC TCC TTG TGA CGG GTT	GCA GAG GTG AGG GAA TGG C
c-KIT	TAC CAA CCA AGG CAG ACA A	CTT TGA GGC AAG GAA CGC
BCL6B	AGG GCA CAG GGA ACT CTT TTC	CCT CCT TTG GCT TGA GTG TTTT

## Results

**Immunocytochemical staining of Sertoli cells and SSCs**

**Figure 1:**
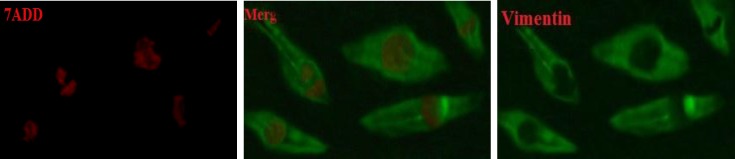
Immunocytochemical staining of the bovine Sertoli cell for Vimentin at day 6 of culture. 7ADD staining is used to demonstrate the nuclei of Sertoli cells (magnification×400).

**Figure 2:**
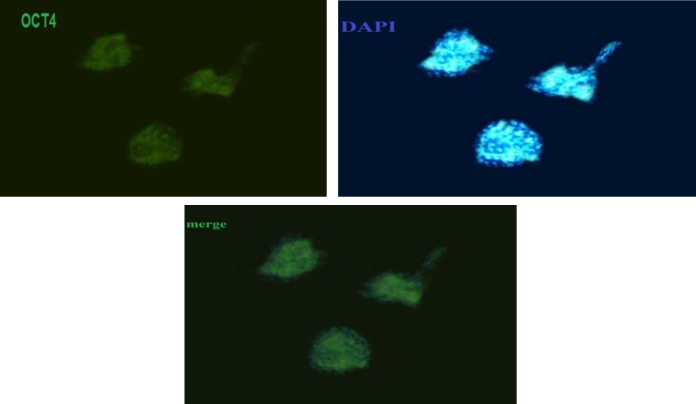
Immunocytochemical staining of bovine SSCs for Oct-4 at day 6 of culture. DAPI is the nuclear staining of SSCs (magnification ×400).

The expression of Vimentin markers detected in the Sertoli cells (Figure 1) and OCT4 was detected in the colonies of SSCs. (Figure 2).

**Numbers of Colonies**

The result of this study showed that the number of colonies in two groups increased until the 12^th^ day of co-culture and after 12 days they decreased. Spermatogonial colonies started to appear around day 3 and increased their number through time. Colonies were counted at days 3, 6, 9, 12 and 15. The number of colonies in the FSH group was significantly higher than that of the control group, during in cell culture (p<0.05).

**Gene Expression**

Expression of FGF2 significantly increased in both group on day 6 and 12 compared to day 0 (p<0.001). Expression of FGF2 was not different in group 2 on day 6 and 12 (p>0.05), while it increased significantly in group 1 during culture (p=0.001). Expression of FGF2 in the FSH group was significantly higher than that of the control group on days 6 and 12 (p<0.05).

Expression of GDNF significantly increased in both groups on days 6 and 12 compared to day 0 (p<0.05); However, it was not different on day 12 compared to day 6 in both groups (p=0.136). On day 6, expression of GDNF in group 2 was higher than group 1 (p=0.001).

Expression of thy1 significantly increased in group 1 on day 6 (p=0.027) and 12 (p<0.0001) compared to day 0 (p<0.05). Expression of thy1 significantly increased on day 12 compared to day 6 in grou1 (p=0.005). Expression of thy1 significantly increased in group 2 on day 6 (p=0.001) and 12 (p<0.0001) compared to day 0 (p<0.01). Moreover, a significant difference was found on day 12 compared to day 6 (p<0.0001). On days 6 and 12, expression of thy1 was higher in group 2 compared to group 1 (p<0.05).

In group 1, expression of C-kit significantly decreased on days 6 and 12 compared to that on day 0 (p<0.05); while no significant difference was found between days 6 and 12 in group 1 (p=0.396). In group 2, expression of C-kit significantly decreased on day 6 (p=0.008) and 12 (p=0.012) compared to day 0 (p<0.05); while no significant difference was found between day 6 and 12 (p=0.365).

In group1, expression of bcl6b significantly increased on day 6 (p=0.004) and 12 (p<0.0001) compared to day 0 (p<0.05). In group 2, expression of bcl6b on day 6 (p=0.005) and 12 (p=0.001) was higher than that on day 0 (p<0.05). However in both groups 1 and 2 no significant difference was found on days 6 and 12 in expression of bcl6b (p>0.05).

**Table IV:** Comparison of colony numbers (mean±SD) between control and experimental groups

**Group**	**Day 3**	**Day 6**	**Day 9**	**Day 12**	**Day 15**
Control	13.75 ± 3.304034	22.00 ± 4.546061	23.75 ± 2.217356	29.00 ± 1.154707	21.00 ± 2.160247
FSH (30 iu/ml)	28.00 ± 5.830952	67.00 ± 4.546061	41.00 ± 9.092121	86.00 ± 10.70825	59.00 ± 4.690416

**Figure 3:**
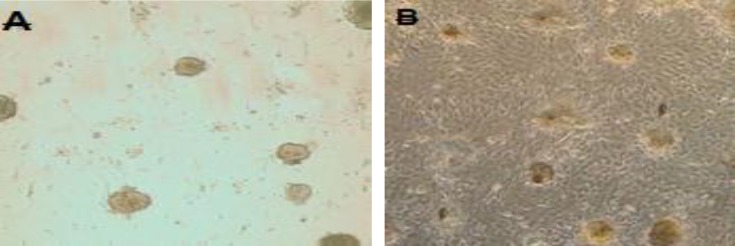
Colonization of SSCs on day 12 in control (A) and FSH (B) groups (magnification×100).

**Figure 4:**
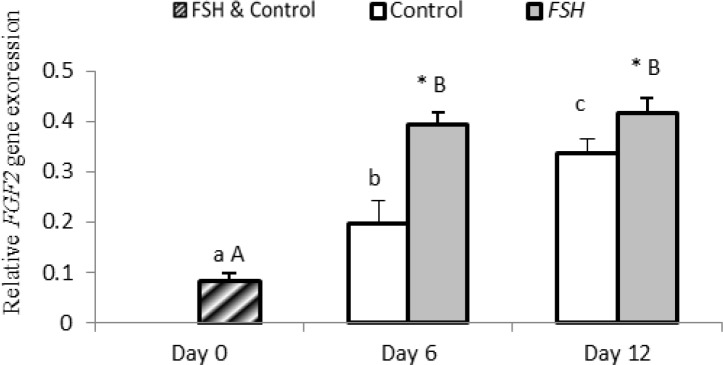
Relative gene expression of FGF2 in control and FSH groups on days 0, 6 and 12. Small letter: indicate difference within control group. Capital letter: indicate difference within FSH group. Different letter [(a.b.c) (A, B)] indicates significant difference within groups in different time-points (p<0.05). * Indicate a significant difference between two experimental groups at the determinate time-points (p<0.05).

**Figure 5:**
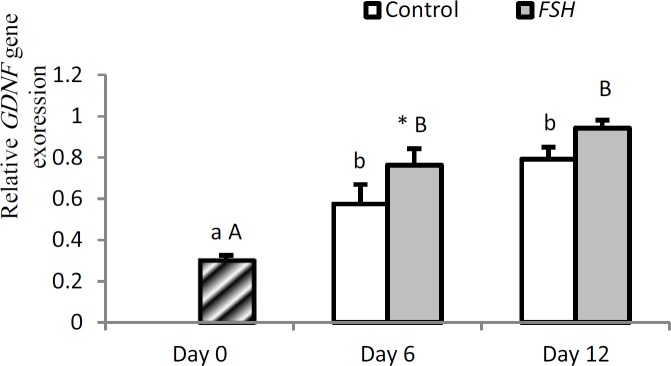
Relative gene expression of GDNF in control and FSH groups on days 0, 6 and 12. Small letter: indicate difference within control group. Capital letter: indicate difference within FSH group. Different letter [(a.b.c) (A, B)] indicates significant difference within groups in different time-points (p<0.05). * indicate a significant difference between two experimental groups at the determinate time-points (p<0.05).

**Figure 6:**
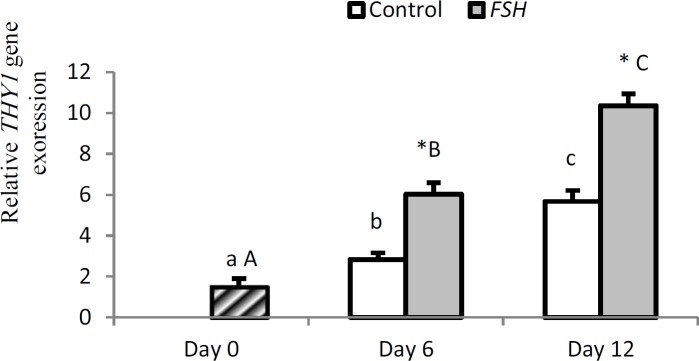
Relative gene expression of thy1 in control and FSH groups on days 0, 6 and 12. Small letter: indicate difference within control group. Capital letter: indicate difference within FSH group. Different letter [(a.b.c) (A, B, C)] indicates significant difference within groups in different time-points (p<0.05). * indicate a significant difference between two experimental groups at the determinate time-points (p<0.05).

**Figure 7:**
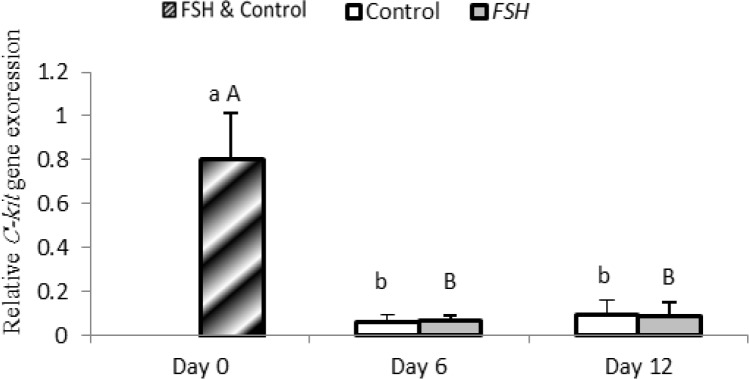
Relative gene expression of C-kit in control and FSH groups on days 0, 6 and 12. Small letter: indicate difference within control group. Capital letter: indicate difference within FSH group. Different letter [(a. b) (A, B)] indicates significant difference within groups in different time-points (p<0.05).

**Figure 8:**
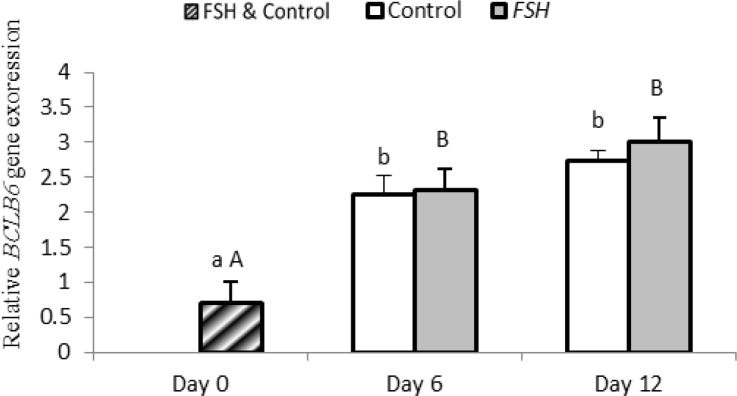
Relative gene expression of bcl6b in control and FSH groups on days 0, 6 and 12. Small letter: indicate difference within control group. Capital letter: indicate difference within FSH group. Different letter [(a.b) (A,B)] indicates significant difference within groups in different time-points (p<0.05).

## Discussion

It seems that FSH stimulates proliferation of Sertoli cells both in vitro and in vivo (27). This hormone is the most important mitogenic factor of Sertoli cells (28). Sertoli cells are stimulated by FSH to secrete growth factors, which stimulate proliferation and colonization of type A SSCs (29). In this study, adding 30 IU/mL FSH to the culture medium has a positive effect on SSC numbers. The number of colonies in FSH treatment group was higher than the control group. This finding is consistent with Anjamrouz *et al* (10). Hence, proliferative SSCs could colonize and preferred proliferation pathway. This pathway could be activated by growth factors produced by Sertoli cells. Vimentin is a cytoskeleton protein in Sertoli cell cytoplasm. For Sertoli cells identification, Vimentin was stained. This finding is similar to the previous studies by Anway *et al* and Tajik *et al* (24, 25). For confirmation of the presence of SSCs, the specific marker Oct-4 was assessed in colonies of SSCs by the method proposed by Kubota *et al* (9). Thy1 is known as SSCs marker in a wide range of mammals (2, 3, 30-32) and as the best marker for enrichment of SSCs (1). 

Moreover, expression of bcl6b (3-5) has been reported in rich populations of SSCs. Increase in thy1 gene expression in response to ordinary culture is consistent with the study performed by Nasiri *et al* (33). On the other hand, C-kit is known as differentiated spermatogonial marker; undifferentiated SSCs are negative in terms of C-kit expression (6). GDNF expression increased in response to ordinary culture and removal of SSCs. Sharp increase in GDNF expression stimulates self-renewal and inhibits differentiation of SSCs (34). On the other hand, undifferentiated SSCs gradually disappear in mice with deficient GDNF gene expression and only Sertoli cells remain in seminiferous tubules of these mice (3). 

In addition, assessment of GDNF expression during different stages of spermatogenesis has shown that GDNF plays a basic role in proliferation and differentiation of SSCs (35). Studies have shown that addition of GDNF in culture leads to proliferation of SSCs (16). Hence, self-renewal of SSCs in ordinary culture can increase expression of GDNF, as previously reported by He *et al* (36). Similar to GDNF, FGF2 stimulated SSCs self-renewal in vitro (9) as well as in vivo (37). Synergistic relationship between FGF2 and GDNF through upregulation of ETS variant (10) (Etv5), increase expression of receptor tyrosine kinas Ret, mediating GDNF signals (37). Therefore, it seems that this effect of FSH for SSCs proliferation could be through upregulation of FGF2 and GDNF in Sertoli cells.

## Conclusion

In conclusion, the present study showed that follicular stimulating hormone (FSH) increases the self-renewal of SSCs and this effect probably mediated through upregulation of GDNF and FGF2 expression in Sertoli cells.
